# Estimating optimum and base selection indices in plant and animal breeding programs by development new and simple SAS and R codes

**DOI:** 10.1038/s41598-023-46368-6

**Published:** 2023-11-03

**Authors:** Mehdi Rahimi, Sandip Debnath

**Affiliations:** 1 Department of Biotechnology, Institute of Science and High Technology and Environmental Sciences, Graduate University of Advanced Technology, Kerman, Iran; 2 Department of Genetics and Plant Breeding, Palli-Siksha Bhavana (Institute of Agriculture), Visva-Bharati University, Sriniketan, West Bengal India

**Keywords:** Agricultural genetics, Plant breeding, Plant sciences, Plant breeding

## Abstract

Selection of desirable genotypes or progenies is perhaps the most important practical method in plant and animal breeding programs. The selection index method is the most useful method to choose superior genotypes based on using simultaneous several traits. The optimum and base selection indices are the two indicators that are most used in plant and animal breeding. In this paper, a simple and practical code was developed for the analysis of optimum, base, and Pesek and Baker selection indices. Four different criteria were used to evaluate the selection index, and the phenotypic and genotypic variance–covariance matrix of traits was obtained based on statistical or genetical design. Moreover, an index that was more efficient on these coefficients was used for the breeding program. The results showed that simultaneous selection for the important traits desired by the breeder through economic values such as heritability, genetic, or phenotypic correlation is the most effective method for selecting the best genotypes. Therefore, the best progeny or genotype can be selected to use in breeding programs. This program provides detailed information on selection indices of segregation and natural populations involving any number of individuals or genotypes. These codes are much easier and simpler than other programs and provide more information than other programs. This code is easy to execute in both R and SAS programs.

## Introduction

The selection of desirable genotypes or progenies is perhaps the most important activity in plant and animal breeding programs. Selection efficiency depends largely on the genetic diversity of the population and the heritability of the studied trait^1^. Selection is often effective in traits with high heritability, compared to those with low heritability. Since one of the important goals in breeding programs is to obtain high-yielding plants and, on the other hand, direct selection for yields and quantitative traits is not very effective because they are controlled by many genes and have low heritability. In most of the correlation studies in plants, it has been determined that yield has a high positive or negative correlation with traits that have high heritability or traits controlled by a small number of genes. Besides, the success of the indirect selection depends mainly on the magnitude and direction (positive or negative association) of the correlation coefficient between the trait of interest and each of the other traits. So, it is better to use indirect selection to improve yields^2^ or other interested traits because selection based on morphological traits with high measurement accuracy and relatively high heritability may be a quick way to screen plant populations and improve yield and quantitative traits and for this reason, using the index can be effective in improving these traits. Also, indices are one of the best methods for simultaneous breeding of traits in breeding programs^3^.

Selection is made for all traits simultaneously by using a total score or index of the net merit of an individual, constructed by combining scores for component characters. Individuals with the highest score are kept for breeding purposes^4^. Since the traits to be considered in selection may not be equally important economically, a type of weighting is required. Unless appropriate weighting is adopted, some traits will receive too much and others too little attention. The amount of weight given to each trait depends on its relative economic value, its heritability, and genetic and phenotypic correlations between different traits^4^.

Choosing a superior progeny in a plant population can affect other progenies because traits are heavily influenced by the environment and often correlate with each other. Therefore, selection based on only one trait to identify superior genotypes may be slightly effective due to low heritability^5,6^. The use of selection indices increases the chances of success of breeding programs because it simultaneously uses different traits to identify the superior genotype. Various information on experimental units is used in selection indices, and the index’s ability is based on a complex economic value of breeder interest traits to increase genetic values^5,6^. The economic return of a crop plant is mostly determined by its several trait values. So, plant breeders study simultaneous selection for those numerous traits that maximize a plant’s economic value. Although the number of traits affects the efficiency of a selection index and the less number of traits has a higher efficiency^7^.

Using some statistical techniques, we can obtain the necessary information for the indirect selection of traits to improve yields. Among these techniques, we can mention the selection index, including optimum and base selection indices^8^. Selection indices have been used in different plants^9–18^. In addition, many studies have been used in animals based on selection indices and these indices have been used to improve and increase performance in them^19–21^.

There are various software applications developed to compute selection indices; although, they do not permit the estimation of some parameters^6,22–26^ such as Genes, MIX, RIndSel, and SelAction. Although these softwares are complete and comprehensive and perform different analysis types and many statistical methods and designs as well as plant breeding method such as diallel analysis, QTL mapping, etc. Besides, these softwares has been widely used by researchers in private and public enterprises, and universities around the world. But some of these softwares that calculate the selection index are complex and do not calculate many of the index evaluation criterias (based on different economic weights) to select the best indices and other programs are not easy and simple in this case.

Thus, due to the lack of easy-to-use specialized software for optimum and base selection indices and their application in plant breeding, in this paper, we described a SAS code developed for the analysis of optimum and base selection indices according to the optimum^27^ and base^28^ selection index methodology, and estimated different criteria for evaluation indices. Simplicity, convenience, and its use in SAS and R softwares are one of the advantages and novelty of this code. Furthermore, the input information of this code can be easily collected by the code written in SAS software ^29^. In addition, there are various criteria to evaluate these two indices in this code, based on which the best index can be selected.

## Materials and methods

### Theory of selection indices

The phenotypic and genotypic variance as well as the covariance between traits were estimated based on the expected value of statistical designs. Then, the broad-sense heritability of traits was calculated based on the formula $h_{b}^{2} = \sigma_{g}^{2} /\sigma_{p}^{2}$ , in which ${\sigma }_{g}^{2}$ and ${\sigma }_{p}^{2}$ are total genetic (or genotypic) and phenotypic variances of each trait, respectively. The phenotypic and genetic correlation coefficients for each pair of traits were calculated using phenotypic and genetic variance and covariance matrices^30^. This input information for this code is easily and simply estimated through the SAS code^29^ and saved in Excel format and can be used for this code in both R and SAS programs. Additionally, the variance–covariance matrix (phenotypic or genotypic) can be obtained by other software as well as Excel. Selection indices based on the studied and used traits in the index (all traits were used in the index) were calculated concerning their phenotypic, genetic, and economic values according to the following equation for the optimum selection index:

1 $$I=\sum {b}_{i}{X}_{i}$$

Here, b_i_ is called the index coefficient (the vector of index coefficients) that is assigned to each trait, and X_i_ is the phenotypic value of each trait as phenotypic trait matrix (n × m). Using the optimum index^31^, the index coefficients were obtained from the following equation:

2 $$b={P}^{-1}Ga$$

In which, b is the vector of index coefficients, P is the phenotypic variance–covariance matrix (m × m), G is the genetic variance–covariance matrix (m × m), and $a$ is the vector of economic values of traits (m × 1) that are assigned by the breeder.

The second index was the base index^28^. If the relative economic values of each trait are determinable, but acceptable and valid estimates of the phenotypic and genetic parameters of the traits are not available, in this case, the use of the base index is recommended for simultaneous improvement of two or more traits. In this method, an index is calculated for each individual using the phenotypic values observed for that trait and by assigning the economic values associated with each trait as the index coefficients:

3 $$I=\sum {a}_{i}{X}_{i}$$

4 $${\text{b}} = {\text{a}}$$

In which, ${a}_{i}$ is the economic value of the trait i and ${X}_{i}$ is also the value of the phenotypic measured for the i-th trait. Also, the vector of index coefficients (b) is equal to the vector of economic values (a) in this index.

However, to assign economic weights to the traits is not a trivial task for breeders, because it demands to know many economic variables of the market (price, objective functions of profit). To avoid this, an index based on the desired genetic gains for each trait was developed by Pešek and Baker ^3,32^.

5 $$I=\sum {b}_{i}{X}_{i}$$

6 $$b={G}^{-1}d$$

where d is the vector of desired genetic gains. The d can be the standard deviation of the genetic variance of the traits or it can be considered as a percentage of the increase or decrease of the traits by the breeder such as study of Pesek and Baker^32^. The amount of increase or decrease of the obtained traits can be considered as d. In this study, the standard deviation of the genetic variance of traits is considered as d.

Four different criteria were used to evaluate the indices. Among the criteria, the correlation coefficients of index and breeding values (R_HI_) were calculated, which would yield the maximum response if this criterion was maximized. Since in addition to grain yield, simultaneous improvement of the genetic value of several traits was the aim, another comparison criterion, namely, genetic gain of total traits ($\Delta H$), was obtained for the index. Each index that has the highest criterion value ($\Delta H$), is the most appropriate in comparison with other indices. Moreover, the expected gain for each trait by the selection index ($\Delta$) was calculated for each trait by the use of the index. The last criterion for evaluating indices was the relative efficiency (RE) of the index compared to direct selection based on the trait (yield). The high proportion of this ratio at the time of using the index means that more genetic gain will be achieved by the yield than direct selection based on yield alone.

In the matrix form, R_HI_ obtained the following relation:

7 $$R_{HI} = \frac{{\sigma_{HI} }}{{\sqrt {\sigma_{I}^{2} \times \sigma_{H}^{2} } }} = \frac{{\sigma_{I} }}{{\sigma_{H} }} = \sqrt {\frac{{\mathop b\limits^{\prime } Ga}}{{\mathop a\limits^{\prime } Ga}}}$$

where $\sigma_{I}^{2}$, $\sigma_{H}^{2}$ and $\sigma_{HI}$ are the variance of index, the variance of breeding value, and covariance of index and breeding value, respectively. P: phenotypic variance–covariance matrix, G: genetic variance–covariance matrix, a: the vector of economic values of traits, b: the vector of index coefficients, and $\mathop a\limits^{\prime }$ and $\mathop b\limits^{\prime }$ are the transpose of the vectors a and b, respectively. In additional, the vector a will be replaced by vector d (the vector of desired gains) for Pesek and Baker index in Eqs. (7) and (8).

The genetic gain of total traits was obtained from the following equation:

8 $$\Delta H = k \times R_{HI} \times \sqrt {\mathop a\limits^{\prime } Ga} = kR_{HI} \sigma_{H}$$

where Selection differential (k) is in standard deviation units and is based on the researcher’s choice of selection intensity (i). If selection intensity(i) is 10%, the value of k is 1.76, ${\sigma }_{H}$: the standard deviation of breeding value, ${R}_{HI}$: the correlation coefficient between the breeding values and index.

The expected genetic advance of each trait based on the index was predicted using Eq. (7).

9 $$\Delta = \frac{kGb}{{\sqrt {\mathop b\limits^{\prime } Pb} }}$$

Relative selection efficiency ratio to direct selection for yield was computed by Eq. (8).

10 $$RE=\frac{{R}_{I}}{{R}_{A}}=\frac{{r}_{G\left(A\right)I}}{{h}_{(A)}}$$

11 $$r_{G\left( A \right)I} = \frac{{\mathop b\limits^{\prime } g}}{{\sqrt {\sigma_{G\left( A \right)}^{2} \times \mathop b\limits^{\prime } Pb} }}\mathop {\lim }\limits_{x \to \infty }$$

where $h_{\left( A \right)}$ is the square root of the broad-sense heritability of the trait A, and $r_{G\left( A \right)I}$ is the correlation between the genotypic values of trait A (trait of interest) and index values, $\sigma_{G\left( A \right)}^{2}$is the genotypic standard deviation (genotypic variance) of trait A, g is the vector of genotypic covariance of trait A (trait of interest) with other traits, and $\mathop a\limits^{\prime }$ and $\mathop b\limits^{\prime }$ are the transpose of the vectors a and b, respectively.

The phenotypic coefficient variation of the index was also calculated from the following relationship:

12 $${CV}_{I}=\left[\frac{{\sigma }_{I}}{\overline{X} }\right]\times 100$$

${\sigma }_{I}:$ the phenotypic standard deviation of the index, $\overline{X }:$ the average of index coefficients obtained for each individual due to the use of the index.

### Description of the SAS and R code

The SAS code (Supplementary Material 1) was written in SAS/IML^33^ and run in SAS^34^. This program is also written in R and can be run in R program code (Supplementary Material 2). This code corresponds to the steps necessary to execute the selection indices according to the optimum and base methods^28,31^.

This code is based on the mathematical derivations presented in optimum and base methods^28,31^. For the analysis to proceed, this code requires an input data file (available at https://www.ebi.ac.uk/biostudies/studies/S-BSST853 as DataFile 3–5) prepared in excel format (CSV). Data can be stored in any format such as xlsx, txt, xls, and others. However, in the proc import section and sub-section (dbms), the format of the data must be specified in the R code, the data format must be specified in the first part of the program and the data introduction. Economic values in the SAS code are entered manually in the code, but in the R code they are stored in an Excel file (available at https://www.ebi.ac.uk/biostudies/studies/S-BSST853 as DataFile 6) and placed in a folder next to the data. The name of the input data file should be changed to the full name (such as DataFile 3-X) in the SAS and R codes. To do these codes, if you do not make any changes to the codes, you must delete the DataFile 3- to 6 in the file names and the file names are changed to X, P, G, and a1.

On file X, there should be phenotypic measurements of traits, on the file P, there should be a phenotypic covariance matrix of traits, and on file G, there must be a genotypic covariance matrix of traits (available at https://www.ebi.ac.uk/biostudies/studies/S-BSST853 as DataFile 3–5). Genotypic and phenotypic covariance matrices are calculated through the mathematical hope of experimental design and can be calculated by the program written for this purpose in SAS^29^.

In the proc import and datafile section, the path and name of the data must be specified (for the X, P, and G data) according to the user of the data. Data for X, P, and G can be stored separately for each file or can be stored in one file on separate sheets. However, the filename or the special sheet of the file should be specified in the proc import section. Users can create a folder on drive C called “selection index’’ and prepare and store data under the same name without the need to change the path of data in codes. Or the data related to these two codes can be placed in a special folder, and at the beginning of the program, the path of this folder, wherever it is on the computer, must be specified for these two codes.

In the proc *IML* section of the SAS code, some information should be provided for the data to be used and should be changed based on user data and the studied trait (Table 1). The information includes the number of genotypes or progeny (NG), the number of studied traits (NT), genetic variance value of trait (w) (trait of interest), selection differential (k), the broad-sense heritability of the interesting trait (h^2^), vector of relative economic values (a1 = {}), and interesting trait number (tr).

**Table 1:**
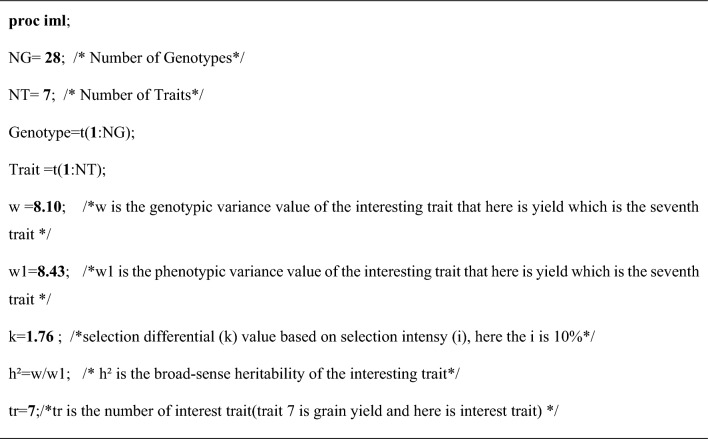
Information needed for use in this code.

Furthermore, for the R code like the SAS code, some parts must be defined before being done. The g is the NT × 1 vector of genotypic variance–covariance of interest trait with other traits (here is the yield which is the seventh trait that is placed in the genotypic or phenotypic matrix) and shown in the genotypic matrix by this G[,7] and 7 is the number of this trait. The wg and wp are the genotypic and phenotypic variance values of the interesting trait (here is the yield which is the seventh trait and 7 is the number of this trait) and shown by these G1[7,7] and P1[7,7], respectively. The results by R code are stored in different sheets of an excel file according to the path to save them and can be accessed. For example, the results when correlations are considered as economic weights are given in Supplementary Material 4 (output_with_EW_corre).

An economic value varies and depends on a researcher’s choice; it can also be any value based on heritability, correlation coefficients, etc. In this research, the amount of economic value (a) was considered in three ways. 1: 1 for all traits, 2: Correlation of traits with yield (Here, the correlation of traits was used based on the DataFile3-X as total correlation. In additional, phenotypic or genotypic correlations calculated based on phenotypic or genotypic variance–covariance matrix (DataFile4-P or DataFile5-G) can also be used), 3: The $\beta$ coefficients of the traits entered in the stepwise regression (Simple stepwise regression is used here by proc reg data = a; model × 7 =  × 1− × 6 / selection = stepwise stb; run;, but stepwise regression by AIC or BIC can be done and selected the best model based on AIC or BIC) model (yield as the dependent variable and considered 1 for it and 0 for non-entered traits) (Table 2). According to the economic values of the traits, different criteria for comparing the indices are given in Table 3 for comparison. In this study, the d vector for Pesek and Baker index was only the standard deviation of the genetic variance of traits of genotypic variance–covariance matrix (The diameter of the genetic variance matrix of the traits). The d vector is calculated in the SAS and R codes based on the command from the genotypic variance–covariance matrix. But the d vector (the d = sqrt(vecdiag(G)) section in SAS code or d = sqrt(diag( G1 )) in R code) can be manually placed in the SAS and R codes based on the opinion of the breeder (different desired genetic gains can be used for d vector) and changed according to a vector section at SAS or R codes).

**Table 2:** Economic weights for calculation of the selection indices.

Traits	Economic weights for optimum and base indices			The d for Pesek and Baker indec
	Method 1	Total correlation	β coefficients	
Plant height	1	− 0.154	0	27.502
Number of grain	1	0.708	0.819	4.554
Number of row	1	0.51	0.725	36.096
Row length	1	0.071	0	0.224
Leaf lenght	1	0.076	0.248	2.484
100-grain weight	1	0.413	0	45.046
Yield	1	1	1	2.846

**Table 3:** Evaluation of different criteria for optimum and base indices based on different economic coefficients.

Index	Economic weights based on Table 2	The expected genetic advance of each trait in index (∆)							${R}_{HI}$	∆H	RE	${CV}_{I}$
		Plant height	Number of grain	Number of row	Row length	Leaf lenght	100-grain weight	Yield				
Optimum	Method 1	0.2115	− 0.3692	54.4801	0.0238	− 1.0277	69.8089	2.6488	0.9887	125.7762	0.5504	13.6215
	Correlation	− 21.9188	− 0.0739	58.9160	0.0458	− 1.5917	73.1789	2.9536	0.9914	66.4291	0.6137	23.2415
	β coefficients	− 16.2316	0.3127	61.4483	0.0355	− 1.1361	61.4314	3.3666	0.9904	47.8910	0.6995	18.6590
Base	Method 1	0.3011	− 0.3384	54.2847	0.0279	− 0.9962	69.7958	2.6734	0.9885	128.6944	0.5555	14.1280
	Correlation	− 21.5200	− 0.0363	58.8330	0.0481	− 1.5666	73.2527	2.9851	0.9912	67.5951	0.6202	24.5539
	β coefficients	− 15.8849	0.3573	61.3100	0.0384	− 1.0991	61.2326	3.4062	0.9900	48.8434	0.7077	20.1070
Pesek and Baker	standard deviation of the genetic variance of traits	9.2366	1.5295	12.1228	0.0751	0.8342	15.1287	0.9559	0.0018	9.2231	0.1986	3.1535

($\Delta$) the expected gain for each trait by the selection index, (*R*_*HI*_) the correlation coefficients of index and breeding value, (∆*H*) the genetic gain of total traits, (*RE*) relative efficiency, (${CV}_{I}$): the phenotypic coefficient variation of index.

The phenotypic value matrix is obtained based on trait evaluation. Moreover, the phenotypic and genotypic variance- covariance matrix of traits is obtained based on statistical or genetical designs in plant and animal breeding programs.

### An example of the SAS and R code used

Data from seven measured traits (quantitative and qualitative traits) on 28 maize inbred lines evaluated in a complete block design with three replications in 2020 at the field were used in this study (DataFile 3). This study complied with relevant institutional, national, and international guidelines and legislation of Iran, and no specific permits were required to collect the plant materials. The phenotypic-genetic covariance matrices of the traits (DataFile 4 and 5) were calculated based on the expected value of statistical designs using the SAS code and saved in Excel format. The PROC IML of SAS was used to estimate the selection index. In the proc iml part, the program needed information including the number of genotypes and traits, genetic variance value of yield, selection intensity, heritability value of yield, NT × 1 vector for relative economic values, and interesting trait number (tr), respectively. The same steps can also be implemented in the R program.

In this study, an economic value (based on Table 2) was used for both optimum and base selection indices, while various economic values could be used based on correlation, heritability, and path coefficients. Then, based on the indices’ evaluation criteria, these two indices were compared together. Finally, the index that was more efficient on these coefficients was used for the breeding program.

## Results and discussion

This code can be easily copied and pasted in the SAS and R softwares and can be used based on user data. In Supplementary Material 3 as well as Table 3, some criteria such as the RHI, ΔH, rG, RE, CV, b values, and Δ of both indices are shown by SAS and R softwares for 28 genotypes and seven traits for the base, optimum and Pesek and Baker indices, respectively. This obtained information can be used to improve maize breeding programs. The selection indices (I) based on the estimated b values for the traits (Supplementary Material 3) are showon below based on Method 1 of economic weights as example:

$$\mathrm{Optimum\, index}=0.963x1+2.150x2+1.119x3-3.851x4+1.610x5+0.976x6-1.411x7$$

$$\mathrm{Base\, index}=x1+x2+x3+x4+x5+x6+x7$$

$$\mathrm{Pesek\, and\, Baker\, index}=0.011x1+5.249x2+0.552x3-3.951x4+3.547x5+0.063x6-9.757x7$$

The coefficient of the traits in the base index is equal to the economic value of the traits. To evaluate the selection strategies for maximizing the maize grain yield, selection indices were calculated based on optimum, base and Pesek and Baker indices with an equal economic trait value (vector a1), as described by Smith^27^ and Brim, et al.^28^. A 10% selection intensity and selection differential (k = 1.76) were used to estimate the expected genetic advance.

The Pesek and Baker index should have been calculated only once because d vector (desired genetic gains) has been used as economic weights. But to see the difference in ranking of genotypes, when the economic weights changed, this index was calculated again. According to the results in Table 3 as well as Supplementary Material 3, the calculated base index had the highest genetic improvement for all the traits (ΔH = 128.69) between the calculated indices when the economic value was one. The selection response of the yield based on the calculated index was RHI = 0. 0.9887 for optimum and was very slightly higher than base index (RHI = 0. 0.9884), although both were much higher than Pesek and Baker index (RHI = 0. 0.0017). This amount indicates that the amount of genetic gain of the yield trait will be 0.9887 if optimum index is used for breeding. RE was calculated to compare the efficiency of the selection index rather than the direct selection of the trait. This value for the optimum (0.5504), base (0.5555) and Pesek and Baker (0.1986) indices indicated that the response to the selection through the index had a lower genetic improvement in the yield compared to the direct self-selection of the yield. If RE is greater than one, this indicates that the response to the selection through the index will be greater for the trait than for the direct self-selection of the trait alone. The base and optimum indices were suitable because RHI, CV, ΔH and RE were higher than the Pesek and Baker index. The coefficients of the index (b) for genotypes are also shown in Supplementary Material 3 for optimum, base and Pesek and Baker indices based on different economic weights. Based on these coefficients, superior genotypes can be selected and used in breeding programs.

The ranking of genetoypes in Table 6 of Supplementary Material 3 shown that the top five genotypes based on optimum index as well as economic weights as Method 1 were genotypes 2, 4, 19, 22 and 6, respectively. While, the genotypes 1, 4, 19, 22 and 6 were identified as five top genotypes, respectively, in base index with the same conditions. In addition, genotypes 3, 17, 8, 16 and 13, respectively were selected as the five top superior genotypes based on the Pesek and Baker index.

Also, for example, the correlation between the indices (based on the index value of the genotypes (I) and the economic weights as Method 1) shown in the Table 7 of Supplementary Material 3. The results showed that correlation between base-optimum, base-Pesek and Baker, and optimum-Pesek and Baker were 0.99979, 0.31516, and 0.32464, respectively. The correlation of base and optimum indices showed that the rankings of genotypes in these two indices were very similar and more than 99% were the same in ranking. Meanwhile, the correlation of index Pesek and Baker with other two indices (base and optimum) showed that the ranking of genotypes based on this index had a high difference with the other two indices.

By taking a glance at the optimum and base indices and comparing them with the Pesek and Baker index based on different economic coefficients (Table 3), we can see that in general the correlation coefficients of the index and breeding value (RHI), the genetic gain of total traits (∆H), relative efficiency (RE) and the phenotypic coefficient variation of the index (${CV}_{I}$) of optimum and base indices are higher than the Pesek and Baker index. Although, the both base and optimum indices were almost close to each other in terms of these criteria, and base index was slightly better than optimum index. In this study, the criterion RHI is almost similar and close for two indices (base and optimum) with three different economic coefficients. But the RE, ∆H and ${CV}_{I}$ criteria were different in these three different methods (Table 3). Considering that these criteria in the base index with β coefficients as economic weight were higher than the others, it can be considered as a superior index. But it should be kept in mind that the index is better when criterion RE is greater than one, in which case simultaneous selection will be better than the selection based on a single interest trait (here yield)^35^. In the base index, the importance of the phenotypic value of each studied trait is directly determined by the factor of economic values, so traits with zero economic value will not be included in the index equation. Additionally, in this index, there is no need to estimate the genetic parameters and the results can be easily obtained and interpreted, so it is preferable to the optimal index.

The goal of plant breeding is the genetic modification of a species in the best possible way. The economic value varies depending on its various traits. Therefore, how to apply selection for several traits to achieve the maximum value of the economy has always been of interest to breeders^8^. Although there is a positive relationship between the yield and the number of its components, the existence of negative relationships between some of the components of the yield has led to the fact that selection for all yield components cannot be used as a factor in increasing the yield^36^. This code has already been used to estimate the optimum and base selection indices at different economic values^37,38^ and it has shown its effectiveness.

Rahimi and Ramezani^38^ examined the base and optimum indices with different economic values (e.g., heritability, path analysis, correlation coefficients of traits with yield) on seven hybrids maize, and finally selected the best index based on the criteria for evaluating the indices. They selected the best genotypes based on the best index. Asghar and Mehdi^39^ reported that the Smith–Hazel and Brim indices were useful for the improvement of a sweet corn population. However, the Brim index was reported to be more efficient than the Smith–Hazel index in genotype improvement for quality traits in a maize population.

To make choices for yield more reliable, breeders need to identify selection criteria that reduce the phenotypic evaluation of traits and focus more on the effect of several traits on the yield. In general, simultaneous selection for the important traits desired by the breeder through economic values such as heritability and genetic or phenotypic correlation is the most effective method for selecting the best genotypes. In this method, an index is defined and the progeny of the population is selected accordingly as a single trait^8^. This code has also been developed based on the selection index of optimum and base methods^28,31^. This code can assist breeders to choose progeny or genotypes because it is simple and convenient and has criteria for comparing different indices based on different economic values. The economic value can be varied and based on this various selection indices can be obtained. Sometimes, multivariate regression coefficients or trait heritability are considered as an economic value. Therefore, this code can compare different selection indices and is chosen as the best selection index. Thus, the best progeny or genotype can be selected to use in breeding programs.

## Conclusion

Considering that the simultaneous use of traits to improve plants and animals can be more beneficial than breeding plants and animals through single traits. Therefore, this simple and practical code can help breeders by simultaneously breed of traits and the use of selection indices in the breeding of plant and animals programs. Moreover, by using different economic values to calculate different indices, an appropriate index can be used for plant or animal breeding programs by comparing the indices according to different criteria. Furthermore, breeders can select superior genotypes based on the coefficient index of each genotype and use them in breeding programs.

### Supplementary Information

Supplementary Information 1.

Supplementary Information 2.

Supplementary Information 3.

Supplementary Information 4.

Supplementary Information 5.

Supplementary Information 6.

Supplementary Tables.

Supplementary Information 7.

## Data Availability

The datasets generated and/or analyzed during the current study are available in the [BioStudies] repository, [https://www.ebi.ac.uk/biostudies/studies/S-BSST853].
