# Supplementary material for: Estimating optimum and base selection indices in plant and animal breeding programs by development new and simple SAS and R codes
Source: Sci Rep. 2023 Nov 3;13:18977. doi: 10.1038/s41598-023-46368-6 (PMC10624857; doi:10.1038/s41598-023-46368-6)
Supplement: Supplementary file 1 — Supplementary Information 1. [file 41598_2023_46368_MOESM1_ESM.docx]

/*Supplementary Material 1- SAS code for Optimum, Base and Base and Pesek Indices*/

DM 'LOG; CLEAR; OUTPUT; CLEAR; ';

Options ps=**5000** ls=**120** nodate nonumber ;

ods graphics off;

/* Import Data */

/*The NG×NT phenotypic value matrix for NG genotypes and NT traits or X matrix*/

**proc** **import** datafile="C:\selection index\Data File 3-6\DataFile3-X.csv" out=X dbms=csv replace;

**run**;

/*The NT×NT phenotypic variance- covariance matrix or P matrix*/

**proc** **import** datafile="C:\selection index\Data File 3-6\DataFile4-P.csv" out=P dbms=csv replace;

**run**;

/*The NT×NT genotypic variance- covariance matrix or G matrix*/

**proc** **import** datafile="C:\selection index\Data File 3-6\DataFile5-G.csv" out=G dbms=csv replace;

**run**;

/* The NT×1 vector of relative economic valuesor a1 Matrix*/

**proc** **import** datafile="C:\selection index\Data File 3-6\DataFile6-a1.csv" out=a1 dbms=csv replace;

**run**;

/* Set working directory */

options dlcreatedir;

libname mydir "C:\selection index";

/* Read data into SAS data sets */

**data** mydir.X;

set X;

**run**;

**data** mydir.P;

set P;

**run**;

**data** mydir.G;

set G;

**run**;

**data** mydir.a1;

set a1;

**run**;

/* Convert data sets to matrices */

**proc** **iml**;

/* Some data that must be entered manually */

NG= **28**; /* Number of Genotypes*/

NT= **7**; /* Number of Traits*/

k = **1.76**;/*selection differential (k) value based on selection intensity (i), here the i is 10%*/

tr=**7**;/*tr is the number of interest trait(trait 7 is grain yield and here is interest trait) */

/* Convert data sets to matrices */

use mydir.X;

read all var _all_ into X;

close mydir.X;

use mydir.P;

read all var _all_ into P;

close mydir.P;

use mydir.G;

read all var _all_ into G;

close mydir.G;

use mydir.a1;

read all var _all_ into a1;

close mydir.a1;

Genotype=t(**1**:NG);

Trait =t(**1**:NT);

wg =G[tr,tr];/*wg is the genotypic variance value of the interest trait that here is yield which is the seventh trait */

wp=P[tr,tr]; /*wp is the phenotypic variance value of the interest trait that here is yield which is the seventh trait */

h2 = wg / wp;/* h² is the broad-sense heritability of the interesting trait, Here, the heritability of the trait of interest was symbolized as h2*/

d = sqrt(VecDiag(G));/* vector of desired gains of traits*/

g1 = G[,tr];/* NT×1 vector of genotypic variance-covariance of interest trait with other traits*/

/* Optimum*/

b_O=inv(P)*G*a1;

I_O=x*b_O;

RHI_O=(sqrt(t(b_O)*P*b_O))/(sqrt(t(a1)*G*a1));

deltaH_O=k*RHI_O*(sqrt(t(a1)*G*a1));

delta_O=(k*G*b_O)/(sqrt(t(b_O)*P*b_O));

rG_O=(t(b_O)*g1)/(sqrt(wg*(t(b_O)*P*b_O)));

RE_O=rG_O/h2;

CV_O=((sqrt (t(b_O)*P*b_O))/((t(I_O)*j(NG,**1**,**1**))/NG))***100**;

**run**;

/* Base*/

b_B=a1;

I_B=x*a1;

RHI_B=(sqrt(t(a1)*G*a1))/(sqrt(t(a1)*P*a1));

deltaH_B=k*sqrt(t(a1)*P*a1);

delta_B=(k*G*a1)/(sqrt(t(a1)*P*a1));

rG_B=(t(a1)*g1)/(sqrt(wg*(t(a1)*P*a1)));

RE_B=rG_B/h2;

CV_B=((sqrt(t(a1)*P*a1))/((t(I_B)*j(NG,**1**,**1**))/NG))***100**;

**run**;

/* Pesek and Baker*/

b_PB=inv(G)*d;

I_PB=x*b_PB;

RHI_PB=(sqrt(t(b_PB)*P*b_PB))/(sqrt(t(d)*G*d));

deltaH_PB=k*RHI_PB*(sqrt(t(d)*G*d));

delta_PB=(k*G*b_PB)/(sqrt(t(b_PB)*P*b_PB));

rG_PB=(t(b_PB)*g1)/(sqrt(wg*(t(b_PB)*P*b_PB)));

RE_PB=rG_PB/h2;

CV_PB=((sqrt (t(b_PB)*P*b_PB))/((t(I_PB)*j(NG,**1**,**1**))/NG))***100**;

**run**;

/* Tables*/

Table1=RHI_O||deltaH_O||rG_O||RE_O||CV_O;

Table2=RHI_B||deltaH_B||rG_B||RE_B||CV_B;

Table3=RHI_PB||deltaH_PB||rG_PB||RE_PB||CV_PB;

Table4=Trait||b_O||delta_O||b_B||delta_B||b_PB||delta_PB;

Table5=Genotype||I_O||I_B||I_PB;

print "Table1: Related parameters of Optimum selection index";

print Table1(|colname={RHI_O deltaH_O rG_O RE_O CV_O}|);

create Table1_1 FROM Table1(|colname={RHI_O deltaH_O rG_O RE_O CV_O}|);

APPEND FROM Table1 ;

print "Table2: Related parameters of Base selection index";

print Table2(|colname={RHI_B deltaH_B rG_B RE_B CV_B}|);

create Table2_1 FROM Table2(|colname={RHI_B deltaH_B rG_B RE_B CV_B}|);

APPEND FROM Table2 ;

print "Table3: Related parameters of Pesek and Baker selection index";

print Table3(|colname={RHI_PB deltaH_PB rG_PB RE_PB CV_PB}|);

create Table3_1 FROM Table3(|colname={RHI_PB deltaH_PB rG_PB RE_PB CV_PB}|);

APPEND FROM Table3 ;

print "Table4: The b values and Expected gain for each trait (delta) for Optimum, Base and, Pesek and Baker selection indices";

print Table4(|colname={Trait b_O delta_O b_B delta_B b_PB delta_PB}|);

create Table4_1 FROM Table4(|colname={Trait b_O delta_O b_B delta_B b_PB delta_PB}|);

APPEND FROM Table4 ;

print "Table5: Coefficients index of genotypes for Optimum (I_O), Base (I_B) and Pesek and Baker (I_PB) selection indices";

PRINT Table5(|colname={Genotype I_O I_B I_PB}|);

create Table5_1 FROM Table5 (|colname={Genotype I_O I_B I_PB}|);

APPEND FROM Table5 ;

**QUIT**;

**proc** **rank** DATA = Table5_1 out=rankings;

var I_O I_B I_PB;

ranks ;

**run**;

title "Table6: The genotypes ranking based on I_O,I_B and I_PB";

**proc** **print** data=rankings;

**run**;

title "Table7: The correlation between I_O,I_B and I_PB ";

**proc** **corr** DATA = Table1_1 out=corr;

var I_O I_B I_PB;

**run**;

**proc** **print** data=corr;

format I_O I_B I_PB **8.6**;

**run**;

/* export data*/

**proc** **export** data=Table1_1 outfile="C:\selection index\SAS-output_with_EW_M1.xlsx" dbms=xlsx replace;

sheet="optimum";

**run**;

**proc** **export** data=Table2_1 outfile="C:\selection index\SAS-output_with_EW_M1.xlsx" dbms=xlsx replace;

sheet="base";

putnames=yes;

**run**;

**proc** **export** data=Table3_1 outfile="C:\selection index\SAS-output_with_EW_M1.xlsx" dbms=xlsx replace;

sheet="PB";

putnames=yes;

**run**;

**proc** **export** data=Table4_1 outfile="C:\selection index\SAS-output_with_EW_M1.xlsx" dbms=xlsx replace;

sheet="b value";

putnames=yes;

**run**;

**proc** **export** data=Table5_1 outfile="C:\selection index\SAS-output_with_EW_M1.xlsx" dbms=xlsx replace;

sheet="Coefficients index";

putnames=yes;

**run**;

**proc** **export** data=rankings outfile="C:\selection index\SAS-output_with_EW_M1.xlsx" dbms=xlsx replace;

sheet="rankings";

putnames=yes;

**run**;

**proc** **export** data=corr outfile="C:\selection index\SAS-output_with_EW_M1.xlsx" dbms=xlsx replace;

sheet="corr";

putnames=yes;

**run**;
