# Supplementary material for: Estimating optimum and base selection indices in plant and animal breeding programs by development new and simple SAS and R codes
Source: Sci Rep. 2023 Nov 3;13:18977. doi: 10.1038/s41598-023-46368-6 (PMC10624857; doi:10.1038/s41598-023-46368-6)
Supplement: Supplementary file 2 — Supplementary Information 2. [file 41598_2023_46368_MOESM2_ESM.docx]

**Supplementary Material 2-** R code for Optimum, Base and Pesek and Baker Indices

#********************************************************************

#* Purpose: estimate optimum, base and Pesek and Baker selection indices to use in plant and animal breeding

#* Author: Mehdi Rahimi

#* Date: 13/10/2023

#* *******************************************************************

# Install required library

install.packages("xlsx")

install.packages("dplyr")

install.packages("corrplot")

# Load required library

library(dplyr)

library(xlsx)

library(corrplot)

#clear env

rm(list = ls())

#set working directory

setwd("C:\\selection index")

#Input manual data

#Number of Genotypes

NG <- 28

#Number of Traits

NT <- 7

#selection differential (k) value based on selection intensity (i), here the i is 10%

k <- 1.76

#tr is the number of interest trait(trait 7 is grain yield and here is interest trait)

tr <- 7

#Data importing and converting to Matrix form

Phenotyping <- read.csv("C:\\selection index\\Data File 3-6\\DataFile3-X.csv", row.names=1)

mat <- as.matrix(Phenotyping)

CovP <- read.csv("C:\\selection index\\Data File 3-6\\DataFile4-P.csv",row.names=1)

mat <- as.matrix(CovP)

CovG <- read.csv("C:\\selection index\\Data File 3-6\\DataFile5-G.csv",row.names=1)

mat <- as.matrix(CovG)

Ecoweight <- read.csv("C:\\selection index\\Data File 3-6\\DataFile6-a1.csv",row.names=1)

mat <- as.matrix(Ecoweight)

X <- data.matrix(Phenotyping)

P<- data.matrix(CovP)

G<- data.matrix(CovG)

a1<- data.matrix(Ecoweight)

Genotype <- 1:NG

Trait <- 1:NT

#wg and wp is the genotypic and phenotypic variance value of the interest trait, respectively. Here is yield which is the seventh trait

wg <- CovG[tr, tr]

wp <- CovP[tr, tr]

#h² is the broad-sense heritability of the interesting trait, Here, the heritability of the trait of interest was symbolized as h2

h2 <- wg / wp

#The g is NT×1 vector of genotypic variance-covariance of interest trait with other traits

g1 <- CovG[, tr]

g <- data.matrix(g1)

#The d is vector of desired gains of traits

d <- sqrt(diag( G ))

#Optimum index (Smith 1936)

b_O<-solve(P)%*%G%*%a1

I_O<-X%*%b_O

RHI_O<-(sqrt(t(b_O)%*%P%*%b_O))/(sqrt(t(a1)%*%G%*%a1))

deltaH_O<-k%*%RHI_O%*%(sqrt(t(a1)%*%G%*%a1))

delta_O <- (k * G %*% b_O)%*%solve((sqrt(t(b_O) %*% P %*% b_O)))

rG_O<-(t(b_O)%*%g)/(sqrt(wg%*%(t(b_O)%*%P%*%b_O)))

RE_O<-rG_O/h2

CV_O<-sqrt (t(b_O)%*%P%*%b_O)/mean(I_O)*100

print(CV_O)

#Base index (Brim et al. 1959)

b_B<-a1

I_B<-X%*%b_B

RHI_B<-(sqrt(t(a1)%*%G%*%a1))/(sqrt(t(a1)%*%P%*%a1))

deltaH_B<-k%*%sqrt(t(a1)%*%P%*%a1)

delta_B<-(k*G%*%a1)%*%solve(sqrt(t(a1)%*%P%*%a1))

rG_B<-(t(a1)%*%g)/(sqrt(wg%*%(t(a1)%*%P%*%a1)))

RE_B<-rG_B/h2

CV_B=sqrt(t(a1)%*%P%*%a1)/mean(I_B)*100

#Pesek and Baker index (Pesek and Baker 1969)

b_PB<-solve(G)%*%d

I_PB<-X%*%b_PB

RHI_PB<-(sqrt(t(b_PB)%*%P%*%b_PB))/(sqrt(t(d)%*%G%*%d))

deltaH_PB<-k%*%RHI_PB%*%(sqrt(t(d)%*%G%*%d))

delta_PB=(k*G%*%b_PB)%*%solve(sqrt(t(b_PB)%*%P%*%b_PB))

rG_PB<-(t(b_PB)%*%g)/(sqrt(wg%*%(t(b_PB)%*%P%*%b_PB)))

RE_PB<-rG_PB/h2

CV_PB<-sqrt (t(b_PB)%*%P%*%b_PB)/mean(I_PB)*100

#Rank and correlation of indices

rO<-rank (I_O)

rB<-rank (I_B)

rPB<-rank (I_PB)

mrank<-cbind(rO,rB,rPB)

head(mrank)

#Output and save results

out1<-cbind(RHI_O,deltaH_O,RE_O,CV_O,RHI_B,deltaH_B,RE_B,CV_B,RHI_PB,deltaH_PB,RE_PB,CV_PB)

colnames(out1) <- c("RHI_O", "deltaH_O", "RE_O", "CV_O","RHI_B", "deltaH_B", "RE_B","CV_B","RHI_PB", "deltaH_PB", "RE_PB", "CV_PB")

out2<-cbind(b_O,delta_O,b_B,delta_B,b_PB,delta_PB)

colnames(out2) <- c("b_O", "delta_O", "b_B", "delta_B","b_PB", "delta_PB")

out3<-cbind(I_O,rO,I_B,rB,I_PB,rPB)

colnames(out3) <- c("I_O", "rO", "I_B", "rB","I_PB", "rPB")

out4<-cor(mrank)

write.xlsx(out1, "C:\\selection index\\R-output_with_EW_M1.xlsx",sheetName="ceritia")

write.xlsx(out2, "C:\\selection index\\R-output_with_EW_M1.xlsx",sheetName="Coefi_index", append=TRUE)

write.xlsx(out3, "C:\\selection index\\R-output_with_EW_M1.xlsx",sheetName="Index_Co_Rank", append=TRUE)

write.xlsx(out4, "C:\\selection index\\R-output_with_EW_M1.xlsx",sheetName="cor", append=TRUE)
