# Supplementary material for: Estimating optimum and base selection indices in plant and animal breeding programs by development new and simple SAS and R codes
Source: Sci Rep. 2023 Nov 3;13:18977. doi: 10.1038/s41598-023-46368-6 (PMC10624857; doi:10.1038/s41598-023-46368-6)
Supplement: Supplementary file 7 — Supplementary Tables. [file 41598_2023_46368_MOESM7_ESM.docx]

| **Output Parameters for Optimum, Base and Pesek and Baker Indices (Economic weights as Method 1)** |
| --- |

| Table1: Related parameters of Optimum selection index |
| --- |

| **Table1** | | | | |
| --- | --- | --- | --- | --- |
| **RHI_O** | **DELTAH_O** | **RG_O** | **RE_O** | **CV_O** |
| 0.9887067 | 125.77616 | 0.5288065 | 0.5503505 | 13.62147 |

| Table2: Related parameters of Base selection index |
| --- |

| **Table2** | | | | |
| --- | --- | --- | --- | --- |
| **RHI_B** | **DELTAH_B** | **RG_B** | **RE_B** | **CV_B** |
| 0.9884876 | 128.6944 | 0.5337124 | 0.5554562 | 14.127987 |

| Table3: Related parameters of Pesek and Baker selection index |
| --- |

| **Table3** | | | | |
| --- | --- | --- | --- | --- |
| **RHI_PB** | **DELTAH_PB** | **RG_PB** | **RE_PB** | **CV_PB** |
| 0.0017573 | 9.2231064 | 0.1908251 | 0.1985994 | 3.1534655 |

| Table4: The b values and Expected gain for each trait (delta) for Optimum, Base and, Pesek and Baker selection indices |
| --- |

| **Table4** | | | | | |
| --- | --- | --- | --- | --- | --- |
| **TRAIT** | **B_O** | **DELTA_O** | **DELTA_B** | **B_PB** | **DELTA_PB** |
| 1 | 0.9629786 | 0.211507 | 0.3011085 | 0.0109093 | 9.2366054 |
| 2 | 2.1496959 | -0.369203 | -0.338416 | 5.2478147 | 1.5295106 |
| 3 | 1.1188804 | 54.480055 | 54.284735 | 0.5520175 | 12.122773 |
| 4 | -3.850599 | 0.0237657 | 0.0279205 | -3.951265 | 0.0750988 |
| 5 | 1.6102579 | -1.027711 | -0.996233 | 3.5465313 | 0.8342394 |
| 6 | 0.9758179 | 69.80893 | 69.795794 | 0.0629345 | 15.128676 |
| 7 | -1.411233 | 2.6488172 | 2.6733909 | -9.756996 | 0.9558519 |

| Table5: Coefficients index of genotypes for Optimum (I_O), Base (I_B) and Pesek and Baker (I_PB) selection indices |
| --- |

| **Table5** | | | |
| --- | --- | --- | --- |
| **GENOTYPE** | **I_O** | **I_B** | **I_PB** |
| 1 | 420.31266 | 409.81 | 160.57457 |
| 2 | 439.32833 | 428.33 | 166.36167 |
| 3 | 550.72477 | 545.28 | 163.24502 |
| 4 | 566.39363 | 561.37 | 165.28622 |
| 5 | 450.67108 | 440.63 | 164.30534 |
| 6 | 442.49173 | 432.69 | 167.23107 |
| 7 | 417.43092 | 409.9 | 161.60656 |
| 8 | 469.96734 | 462.73 | 163.70837 |
| 9 | 483.30656 | 475.61 | 172.77388 |
| 10 | 563.27642 | 556.81 | 166.55932 |
| 11 | 557.09647 | 551.43 | 167.3985 |
| 12 | 569.13596 | 563.69 | 162.49655 |
| 13 | 606.44134 | 604.22 | 164.90863 |
| 14 | 508.86366 | 501.94 | 162.3941 |
| 15 | 717.09895 | 712.45 | 172.39908 |
| 16 | 547.96026 | 543.37 | 163.61758 |
| 17 | 513.235 | 507.28 | 160.38069 |
| 18 | 428.54048 | 417.55 | 179.9942 |
| 19 | 522.89771 | 514.99 | 164.91631 |
| 20 | 495.07969 | 488.28 | 162.72527 |
| 21 | 509.16538 | 503.9 | 163.76304 |
| 22 | 531.76281 | 524.96 | 163.25664 |
| 23 | 518.42666 | 511.71 | 166.89988 |
| 24 | 613.64307 | 608.98 | 170.09336 |
| 25 | 478.31079 | 468.37 | 157.51016 |
| 26 | 535.68617 | 526.77 | 167.78279 |
| 27 | 605.46943 | 598.91 | 172.65072 |
| 28 | 627.21295 | 619.92 | 178.17593 |

| Table6: The genotypes ranking based on I_O,I_B and I_PB |
| --- |

| **Obs** | **GENOTYPE** | **I_O** | **I_B** | **I_PB** |
| --- | --- | --- | --- | --- |
| **1** | 1 | 2 | 1 | 3 |
| **2** | 2 | 4 | 4 | 17 |
| **3** | 3 | 19 | 19 | 8 |
| **4** | 4 | 22 | 22 | 16 |
| **5** | 5 | 6 | 6 | 13 |
| **6** | 6 | 5 | 5 | 20 |
| **7** | 7 | 1 | 2 | 4 |
| **8** | 8 | 7 | 7 | 11 |
| **9** | 9 | 9 | 9 | 26 |
| **10** | 10 | 21 | 21 | 18 |
| **11** | 11 | 20 | 20 | 21 |
| **12** | 12 | 23 | 23 | 6 |
| **13** | 13 | 25 | 25 | 14 |
| **14** | 14 | 11 | 11 | 5 |
| **15** | 15 | 28 | 28 | 24 |
| **16** | 16 | 18 | 18 | 10 |
| **17** | 17 | 13 | 13 | 2 |
| **18** | 18 | 3 | 3 | 28 |
| **19** | 19 | 15 | 15 | 15 |
| **20** | 20 | 10 | 10 | 7 |
| **21** | 21 | 12 | 12 | 12 |
| **22** | 22 | 16 | 16 | 9 |
| **23** | 23 | 14 | 14 | 19 |
| **24** | 24 | 26 | 26 | 23 |
| **25** | 25 | 8 | 8 | 1 |
| **26** | 26 | 17 | 17 | 22 |
| **27** | 27 | 24 | 24 | 25 |
| **28** | 28 | 27 | 27 | 27 |

| Table7: The correlation between I_O,I_B and I_PB |
| --- |

The CORR Procedure

| **3 Variables:** | I_O I_B I_PB |
| --- | --- |

| **Simple Statistics** | | | | | | |
| --- | --- | --- | --- | --- | --- | --- |
| **Variable** | **N** | **Mean** | **Std Dev** | **Sum** | **Minimum** | **Maximum** |
| **I_O** | 28 | 524.64037 | 70.93640 | 14690 | 417.43092 | 717.09895 |
| **I_B** | 28 | 517.56714 | 72.56168 | 14492 | 409.81000 | 712.45000 |
| **I_PB** | 28 | 166.17912 | 5.17867 | 4653 | 157.51016 | 179.99420 |

| **Pearson Correlation Coefficients, N = 28  Prob > \|r\| under H0: Rho=0** | | | |
| --- | --- | --- | --- |
|  | **I_O** | **I_B** | **I_PB** |
| **I_O** | \| 1.00000 \| \| --- \| \|  \| | \| 0.99979 \| \| --- \| \| <.0001 \| | \| 0.32464 \| \| --- \| \| 0.0919 \| |
| **I_B** | \| 0.99979 \| \| --- \| \| <.0001 \| | \| 1.00000 \| \| --- \| \|  \| | \| 0.31516 \| \| --- \| \| 0.1023 \| |
| **I_PB** | \| 0.32464 \| \| --- \| \| 0.0919 \| | \| 0.31516 \| \| --- \| \| 0.1023 \| | \| 1.00000 \| \| --- \| \|  \| |

| **Output Parameters for Optimum, Base and Pesek and Baker Indices (Economic weights as Correlation)** |
| --- |

| Table1: Related parameters of Optimum selection index |
| --- |

| **Table1** | | | | |
| --- | --- | --- | --- | --- |
| **RHI_O** | **DELTAH_O** | **RG_O** | **RE_O** | **CV_O** |
| 0.9914347 | 66.429089 | 0.589647 | 0.6136697 | 23.241502 |

| Table2: Related parameters of Base selection index |
| --- |

| **Table2** | | | | |
| --- | --- | --- | --- | --- |
| **RHI_B** | **DELTAH_B** | **RG_B** | **RE_B** | **CV_B** |
| 0.9912399 | 67.595134 | 0.5959437 | 0.6202229 | 24.553871 |

| Table3: Related parameters of Pesek and Baker selection index |
| --- |

| **Table3** | | | | |
| --- | --- | --- | --- | --- |
| **RHI_PB** | **DELTAH_PB** | **RG_PB** | **RE_PB** | **CV_PB** |
| 0.0017573 | 9.2231064 | 0.1908251 | 0.1985994 | 3.1534655 |

| Table4: The b values and Expected gain for each trait (delta) for Optimum, Base and, Pesek and Baker selection indices |
| --- |

| **Table4** | | | | | |
| --- | --- | --- | --- | --- | --- |
| **TRAIT** | **B_O** | **DELTA_O** | **DELTA_B** | **B_PB** | **DELTA_PB** |
| 1 | -0.169547 | -21.91876 | -21.51996 | 0.0109093 | 9.2366054 |
| 2 | 1.2614609 | -0.07388 | -0.036328 | 5.2478147 | 1.5295106 |
| 3 | 0.5667771 | 58.916004 | 58.833041 | 0.5520175 | 12.122773 |
| 4 | -1.859062 | 0.0458267 | 0.0481473 | -3.951265 | 0.0750988 |
| 5 | 0.4124795 | -1.591718 | -1.566608 | 3.5465313 | 0.8342394 |
| 6 | 0.4048206 | 73.178915 | 73.252709 | 0.0629345 | 15.128676 |
| 7 | -0.19543 | 2.9535701 | 2.9851104 | -9.756996 | 0.9558519 |

| Table5: Coefficients index of genotypes for Optimum (I_O), Base (I_B) and Pesek and Baker (I_PB) selection indices |
| --- |

| **Table5** | | | |
| --- | --- | --- | --- |
| **GENOTYPE** | **I_O** | **I_B** | **I_PB** |
| 1 | 108.25104 | 100.75932 | 160.57457 |
| 2 | 120.13276 | 112.51932 | 166.36167 |
| 3 | 157.4909 | 152.28573 | 163.24502 |
| 4 | 163.53049 | 158.45888 | 165.28622 |
| 5 | 122.45364 | 115.185 | 164.30534 |
| 6 | 115.76029 | 108.57619 | 167.23107 |
| 7 | 101.42608 | 95.0398 | 161.60656 |
| 8 | 118.36739 | 112.12513 | 163.70837 |
| 9 | 117.24177 | 110.51696 | 172.77388 |
| 10 | 204.92938 | 199.21346 | 166.55932 |
| 11 | 170.91561 | 165.51958 | 167.3985 |
| 12 | 158.36963 | 153.31264 | 162.49655 |
| 13 | 188.21889 | 184.22023 | 164.90863 |
| 14 | 178.57409 | 172.87584 | 162.3941 |
| 15 | 250.94761 | 246.15884 | 172.39908 |
| 16 | 188.38304 | 183.44283 | 163.61758 |
| 17 | 180.24254 | 175.04612 | 160.38069 |
| 18 | 116.77002 | 109.01395 | 179.9942 |
| 19 | 176.69976 | 170.38287 | 164.91631 |
| 20 | 173.8975 | 168.0231 | 162.72527 |
| 21 | 139.92614 | 134.66499 | 163.76304 |
| 22 | 179.72879 | 173.92917 | 163.25664 |
| 23 | 190.81803 | 185.22404 | 166.89988 |
| 24 | 198.48553 | 193.68461 | 170.09336 |
| 25 | 165.30978 | 158.16163 | 157.51016 |
| 26 | 142.17968 | 135.51591 | 167.78279 |
| 27 | 203.57409 | 197.65422 | 172.65072 |
| 28 | 214.52753 | 208.15402 | 178.17593 |

| Table6: The genotypes ranking based on I_O,I_B and I_PB |
| --- |

| **Obs** | **GENOTYPE** | **I_O** | **I_B** | **I_PB** |
| --- | --- | --- | --- | --- |
| **1** | 1 | 2 | 2 | 3 |
| **2** | 2 | 7 | 7 | 17 |
| **3** | 3 | 11 | 11 | 8 |
| **4** | 4 | 13 | 14 | 16 |
| **5** | 5 | 8 | 8 | 13 |
| **6** | 6 | 3 | 3 | 20 |
| **7** | 7 | 1 | 1 | 4 |
| **8** | 8 | 6 | 6 | 11 |
| **9** | 9 | 5 | 5 | 26 |
| **10** | 10 | 26 | 26 | 18 |
| **11** | 11 | 15 | 15 | 21 |
| **12** | 12 | 12 | 12 | 6 |
| **13** | 13 | 21 | 22 | 14 |
| **14** | 14 | 18 | 18 | 5 |
| **15** | 15 | 28 | 28 | 24 |
| **16** | 16 | 22 | 21 | 10 |
| **17** | 17 | 20 | 20 | 2 |
| **18** | 18 | 4 | 4 | 28 |
| **19** | 19 | 17 | 17 | 15 |
| **20** | 20 | 16 | 16 | 7 |
| **21** | 21 | 9 | 9 | 12 |
| **22** | 22 | 19 | 19 | 9 |
| **23** | 23 | 23 | 23 | 19 |
| **24** | 24 | 24 | 24 | 23 |
| **25** | 25 | 14 | 13 | 1 |
| **26** | 26 | 10 | 10 | 22 |
| **27** | 27 | 25 | 25 | 25 |
| **28** | 28 | 27 | 27 | 27 |

| Table7: The correlation between I_O,I_B and I_PB |
| --- |

The CORR Procedure

| **3 Variables:** | I_O I_B I_PB |
| --- | --- |

| **Simple Statistics** | | | | | | |
| --- | --- | --- | --- | --- | --- | --- |
| **Variable** | **N** | **Mean** | **Std Dev** | **Sum** | **Minimum** | **Maximum** |
| **I_O** | 28 | 162.39829 | 37.53251 | 4547 | 101.42608 | 250.94761 |
| **I_B** | 28 | 156.41659 | 38.18184 | 4380 | 95.03980 | 246.15884 |
| **I_PB** | 28 | 166.17912 | 5.17867 | 4653 | 157.51016 | 179.99420 |

| **Pearson Correlation Coefficients, N = 28  Prob > \|r\| under H0: Rho=0** | | | |
| --- | --- | --- | --- |
|  | **I_O** | **I_B** | **I_PB** |
| **I_O** | \| 1.00000 \| \| --- \| \|  \| | \| 0.99981 \| \| --- \| \| <.0001 \| | \| 0.20280 \| \| --- \| \| 0.3007 \| |
| **I_B** | \| 0.99981 \| \| --- \| \| <.0001 \| | \| 1.00000 \| \| --- \| \|  \| | \| 0.19574 \| \| --- \| \| 0.3182 \| |
| **I_PB** | \| 0.20280 \| \| --- \| \| 0.3007 \| | \| 0.19574 \| \| --- \| \| 0.3182 \| | \| 1.00000 \| \| --- \| \|  \| |

| **Output Parameters for Optimum, Base and Pesek and Baker Indices (Economic weights as ß coefficients)** |
| --- |

| Table1: Related parameters of Optimum selection index |
| --- |

| **Table1** | | | | |
| --- | --- | --- | --- | --- |
| **RHI_O** | **DELTAH_O** | **RG_O** | **RE_O** | **CV_O** |
| 0.990358 | 47.890991 | 0.6721074 | 0.6994896 | 18.659044 |

| Table2: Related parameters of Base selection index |
| --- |

| **Table2** | | | | |
| --- | --- | --- | --- | --- |
| **RHI_B** | **DELTAH_B** | **RG_B** | **RE_B** | **CV_B** |
| 0.990047 | 48.843386 | 0.6800056 | 0.7077096 | 20.107003 |

| Table3: Related parameters of Pesek and Baker selection index |
| --- |

| **Table3** | | | | |
| --- | --- | --- | --- | --- |
| **RHI_PB** | **DELTAH_PB** | **RG_PB** | **RE_PB** | **CV_PB** |
| 0.0017573 | 9.2231064 | 0.1908251 | 0.1985994 | 3.1534655 |

| Table4: The b values and Expected gain for each trait (delta) for Optimum, Base and, Pesek and Baker selection indices |
| --- |

| **Table4** | | | | | |
| --- | --- | --- | --- | --- | --- |
| **TRAIT** | **B_O** | **DELTA_O** | **DELTA_B** | **B_PB** | **DELTA_PB** |
| 1 | -0.012359 | -16.23159 | -15.88489 | 0.0109093 | 9.2366054 |
| 2 | 1.3846687 | 0.3126865 | 0.3572984 | 5.2478147 | 1.5295106 |
| 3 | 0.7731881 | 61.448333 | 61.309954 | 0.5520175 | 12.122773 |
| 4 | -1.807612 | 0.0354678 | 0.038418 | -3.951265 | 0.0750988 |
| 5 | 0.5814885 | -1.136126 | -1.099082 | 3.5465313 | 0.8342394 |
| 6 | 0.0052166 | 61.431437 | 61.232627 | 0.0629345 | 15.128676 |
| 7 | -0.219866 | 3.3666182 | 3.4061807 | -9.756996 | 0.9558519 |

| Table5: Coefficients index of genotypes for Optimum (I_O), Base (I_B) and Pesek and Baker (I_PB) selection indices |
| --- |

| **Table5** | | | |
| --- | --- | --- | --- |
| **GENOTYPE** | **I_O** | **I_B** | **I_PB** |
| 1 | 110.3702 | 101.56311 | 160.57457 |
| 2 | 119.86458 | 110.814 | 166.36167 |
| 3 | 150.2833 | 143.34852 | 163.24502 |
| 4 | 146.57233 | 139.40787 | 165.28622 |
| 5 | 122.70756 | 114.06126 | 164.30534 |
| 6 | 120.19398 | 111.6551 | 167.23107 |
| 7 | 106.75232 | 98.98067 | 161.60656 |
| 8 | 119.95851 | 112.19483 | 163.70837 |
| 9 | 93.375282 | 84.10282 | 172.77388 |
| 10 | 167.70628 | 159.90426 | 166.55932 |
| 11 | 145.34766 | 137.68536 | 167.3985 |
| 12 | 152.33328 | 145.49969 | 162.49655 |
| 13 | 149.94748 | 143.28383 | 164.90863 |
| 14 | 157.18147 | 149.79869 | 162.3941 |
| 15 | 221.30188 | 214.53108 | 172.39908 |
| 16 | 171.00488 | 164.46026 | 163.61758 |
| 17 | 160.93869 | 154.10574 | 160.38069 |
| 18 | 123.40959 | 114.3057 | 179.9942 |
| 19 | 156.41824 | 148.4714 | 164.91631 |
| 20 | 151.94843 | 144.44744 | 162.72527 |
| 21 | 134.10688 | 127.10812 | 163.76304 |
| 22 | 156.58815 | 149.03234 | 163.25664 |
| 23 | 159.80291 | 152.21943 | 166.89988 |
| 24 | 164.07512 | 156.73377 | 170.09336 |
| 25 | 122.46072 | 112.98718 | 157.51016 |
| 26 | 138.01844 | 129.57699 | 167.78279 |
| 27 | 178.29455 | 170.45672 | 172.65072 |
| 28 | 182.32325 | 173.85712 | 178.17593 |

| Table6: The genotypes ranking based on I_O,I_B and I_PB |
| --- |

| **Obs** | **GENOTYPE** | **I_O** | **I_B** | **I_PB** |
| --- | --- | --- | --- | --- |
| **1** | 1 | 3 | 3 | 3 |
| **2** | 2 | 4 | 4 | 17 |
| **3** | 3 | 15 | 15 | 8 |
| **4** | 4 | 13 | 13 | 16 |
| **5** | 5 | 8 | 8 | 13 |
| **6** | 6 | 6 | 5 | 20 |
| **7** | 7 | 2 | 2 | 4 |
| **8** | 8 | 5 | 6 | 11 |
| **9** | 9 | 1 | 1 | 26 |
| **10** | 10 | 24 | 24 | 18 |
| **11** | 11 | 12 | 12 | 21 |
| **12** | 12 | 17 | 17 | 6 |
| **13** | 13 | 14 | 14 | 14 |
| **14** | 14 | 20 | 20 | 5 |
| **15** | 15 | 28 | 28 | 24 |
| **16** | 16 | 25 | 25 | 10 |
| **17** | 17 | 22 | 22 | 2 |
| **18** | 18 | 9 | 9 | 28 |
| **19** | 19 | 18 | 18 | 15 |
| **20** | 20 | 16 | 16 | 7 |
| **21** | 21 | 10 | 10 | 12 |
| **22** | 22 | 19 | 19 | 9 |
| **23** | 23 | 21 | 21 | 19 |
| **24** | 24 | 23 | 23 | 23 |
| **25** | 25 | 7 | 7 | 1 |
| **26** | 26 | 11 | 11 | 22 |
| **27** | 27 | 26 | 26 | 25 |
| **28** | 28 | 27 | 27 | 27 |

| Table7: The correlation between I_O,I_B and I_PB |
| --- |

The CORR Procedure

| **3 Variables:** | I_O I_B I_PB |
| --- | --- |

| **Simple Statistics** | | | | | | |
| --- | --- | --- | --- | --- | --- | --- |
| **Variable** | **N** | **Mean** | **Std Dev** | **Sum** | **Minimum** | **Maximum** |
| **I_O** | 28 | 145.83164 | 27.04116 | 4083 | 93.37528 | 221.30188 |
| **I_B** | 28 | 138.02119 | 27.56773 | 3865 | 84.10282 | 214.53108 |
| **I_PB** | 28 | 166.17912 | 5.17867 | 4653 | 157.51016 | 179.99420 |

| **Pearson Correlation Coefficients, N = 28  Prob > \|r\| under H0: Rho=0** | | | |
| --- | --- | --- | --- |
|  | **I_O** | **I_B** | **I_PB** |
| **I_O** | \| 1.00000 \| \| --- \| \|  \| | \| 0.99970 \| \| --- \| \| <.0001 \| | \| 0.24288 \| \| --- \| \| 0.2130 \| |
| **I_B** | \| 0.99970 \| \| --- \| \| <.0001 \| | \| 1.00000 \| \| --- \| \|  \| | \| 0.23046 \| \| --- \| \| 0.2381 \| |
| **I_PB** | \| 0.24288 \| \| --- \| \| 0.2130 \| | \| 0.23046 \| \| --- \| \| 0.2381 \| | \| 1.00000 \| \| --- \| \|  \| |
